# Perampanel and Challenging Behaviour in Intellectual Disability and Epilepsy: A Management Dilemma

**DOI:** 10.1155/2014/409209

**Published:** 2014-12-17

**Authors:** Emily Dolton, Ansar Choudry

**Affiliations:** ^1^CT2 West Midlands Deanery, Dudley and Walsall Mental Health, Bloxwich Hospital, Bloxwich, Walsall WS3 2JJ, UK; ^2^Heath Lane Hospital, Black Country Partnership Trust, West Bromwich B71 2BG, UK

## Abstract

We describe a case of a patient with a diagnosis of moderate learning disability with challenging behaviour and treatment refractory epilepsy. Antiepileptics can increase challenging behaviour; however, antipsychotics can provoke seizures. This results in a difficult balance for patient care. Due to worsening seizures, the patient was prescribed perampanel. This increased her aggression and agitation resulting in admission. We trialled four antipsychotic drugs to reduce her challenging behaviour, two of which worsened her seizures. It was necessary to continue antiepileptic medication to maintain adequate seizure control. However, the resulting uncontrolled challenging behaviour persisted, meaning she was unable to return to her family home on discharge. This case emphasises the difficult scenario clinician's encounter when balancing the use of antipsychotics and antiepileptics. The case demonstrates the significant functional loss due to challenging behaviour, balanced against controlling life threatening seizures.

## 1. Introduction

Perampanel was introduced to the UK market in 2012. Perampanel has been evaluated and found to be effective primarily in the use of treatment refractory epilepsy [1]. Perampanel is a highly selective, noncompetitive AMPA-type glutamate receptor antagonist and is the first antiepileptic medication with this mechanism of action. However, perampanel, along with other antiepileptics, has side effects of several psychiatric symptoms including aggression, anger, anxiety, confusion, irritability, and mood alteration. The incidence of such symptoms varies between less than 1% for anger at 4 mg daily dose and 12% for irritability at 12 mg daily dose [2].

It is well established that epilepsy is more prevalent in adults and children with intellectual disabilities [3]. Many require life-long antiepileptic medication for adequate seizure control. However, challenging behaviour such as self-injury and aggression is also common in intellectual disabilities. Challenging behaviour is behaviour which may put the patient or others at risk or which may prevent the use of ordinary community facilities or a normal home life. This would most commonly include physical aggression towards others, self-injurious behaviour, damage to property, and lack of awareness of danger. Challenging behaviour is usually due to an unfulfilled need or communication issues. Studies show that approximately 10–20% of those with intellectual disabilities exhibit challenging behavior [4]. It is important to highlight the significant overlap between symptoms of challenging behaviour and the known side effects of perampanel and other antiepileptics.

There are various psychological treatment methods for such behaviours. However, sometimes medication is used for symptom control, often in the form of antipsychotics [5]. Antipsychotics may, in some patients, lower seizure threshold [6]. Some antiepileptics can, in turn, increase challenging behaviour. This creates a difficult management situation for learning disability psychiatrists. It is equally important for general practitioners and specialists prescribing antiepileptics to be aware of the challenges that exist in managing patients with intellectual disabilities.

## 2. Case Presentation

A 37-year-old female, with a background of Tourette's syndrome, moderate learning disability with challenging behaviour, and treatment refractory epilepsy, was prescribed perampanel, for poor seizure control, in November 2012 by her neuropsychiatrist. This was gradually increased to 8 mg. This was as an adjunct to three antiepileptics: gabapentin, zonisamide, and sodium valproate. The patient has suffered with idiopathic epilepsy with generalised seizures since childhood. She has been tried on several different antiepileptics over the years and at this time was taking a combination of these three. However, still she continued to have tonic-clonic seizures at a frequency of approximately six-eight per month and lasted up to 15 minutes. Her epilepsy had previously been managed by her learning disability psychiatry team; however, due to the resistance of her seizures to medication she was referred to the neuropsychiatry team. An important factor in her epilepsy management was that her seizures had been so severe and prolonged that she had lost the vision in one eye, due to traumatic injury. Therefore, both specialists were keen to avoid any potential visual side effects noted with some antiepileptics (e.g., vigabatrin). It was concluded that perampanel would be an appropriate choice.

In April 2013 she presented with verbal and physical aggression towards her parents and self-injurious behaviours such as throwing herself against walls. Her parents reported that this change in her behaviour had occurred since her perampanel was increased to 8 mg daily. Her seizure significantly decreased in both frequency and duration. She was under the care of learning disability outpatients clinics and therefore Behavioural Support Team intervention was arranged. This consists of a specialist team of nursing staff, who are trained in behavioural psychological techniques and have the capacity to visit her home to address environmental factors. They were involved in her case for a number of weeks and saw no improvement in her behaviour. The severity of her physical aggression towards others, in particular her mother, increased—including kicking and hitting her. Her parents no longer felt able to cope with her living at home. Global clinical impression was that the patient was severely ill and had become worse over these weeks. Therefore the decision was made between the medical team, Behavioural Support Team, and family to admit her to a learning disability psychiatric unit. The patient at this point in time did not have the capacity to consent to admission but was eventually admitted informally.

Prior to presentation, her mental state had been stable on quetiapine 175 mg twice daily for the past 10 years. She had one previous admission due to challenging behaviour and poor seizure control, in 2001. At this time she was taking sulpiride 200 mg twice daily to control tics associated with her Tourette's syndrome. It is important to note that, in this case, the patient had a past history of significant challenging behaviour symptoms and was therefore vulnerable to the side effects of the drug discussed. However, her long period of stability with respect to her challenging behaviour symptoms during periods with severe and frequent seizures indicates that it is unlikely to be a symptom of the epilepsy itself.

During this admission, quetiapine was gradually increased to 350 mg twice daily with no improvement in her challenging behaviour. She was reviewed by her neuropsychiatrist who reduced her perampanel to 6 mg; however her behaviours remained the same. Risperidone (2 mg twice daily) was trialled and then amisulpride (200 mg twice daily) separately, which successfully reduced her challenging behaviour. However both medications increased both the frequency and duration of her seizures. Therefore perampanel 8 mg was reinstated. The amisulpride dose was reduced to 50 mg twice daily; however prolonged seizures persisted. At this point in time, she was unable to attend her day centre, due to her agitation and physical aggression whilst in the transport vehicle. It was therefore deemed that she would be unable to tolerate an EEG to investigate any qualitative changes in her seizures.

Zuclopenthixol was commenced for behavioural control, with little effect on her seizures, but also little effect on her mental state. After further discussion with her neuropsychiatrist, perampanel was gradually decreased to 4 mg. She suffered no consequent seizures; however there was no improvement in challenging behaviour. Due to significant extrapyramidal side effects whilst taking zuclopenthixol it was discontinued.

She was subsequently commenced on aripiprazole and challenging behaviour began to show some signs of improvement with minimal increase in her seizure activity. However, she still required inpatient care and close monitoring. Due to her challenging behaviour during leave to her parent's house, she was unable to return to her family home upon discharge. Therefore a supported living placement was identified. She was also excluded from day care due to significant disruptions caused by her agitation. The effect on her social life and her family's has therefore been substantial.

## 3. Discussion

In conclusion, as with the aetiology of any psychiatric case, there are likely to be various relevant biopsychosocial factors. Her past history of challenging behaviour and learning disability were important predisposing factors. However, it does seem likely that, in this case, commencing perampanel was an important precipitating factor. In particular her symptoms were noted by her parents at the time of the increase in medication. Despite decreases in perampanel, behaviours persisted, though it effectively controlled seizures.

The current literature demonstrates an increase in challenging behaviour with perampanel but does not describe the functional loss seen in this case. This loss can be difficult to restore and has an impact on both patient and family. This case also emphasises the balance of managing seizure control and challenging behaviour and the difficulty encountered. This balance can involve more than one team of specialists in order to provide the best care possible for the patient (neuropsychiatry and learning disability psychiatry). As clinicians, often the risks of morbidity associated with physical conditions are justifiably prioritised over optimal mental health. However, it is important to attempt to maximise both physical and mental health wherever possible.

In summary there are several learning points highlighted in this case. Firstly, antiepileptics should be prescribed with caution in patients with intellectual disabilities and a past history of challenging behaviour. Also, once patients present with challenging behaviour it may be difficult to successfully restore their level of functioning. This can have a significant impact on both the patient and their family. Finally, both learning disability psychiatrists and neuropsychiatrists encounter difficult clinical scenarios, involving a balancing act between the use of antipsychotics and the use of antiepileptics in the management of epilepsy and challenging behaviour.
